# Survey of methanotrophic diversity in various ecosystems by degenerate methane monooxygenase gene primers

**DOI:** 10.1186/s13568-017-0466-2

**Published:** 2017-08-23

**Authors:** Mohammad Ghashghavi, Mike S. M. Jetten, Claudia Lüke

**Affiliations:** Department of Microbiology, Radboud University, Heijendaalsweg 135, 6525 AJ Nijmegen, The Netherlands

**Keywords:** Methane, Particulate methane monooxygenase, Diversity, Methanotroph, Genetic marker

## Abstract

Methane is the second most important greenhouse gas contributing to about 20% of global warming. Its mitigation is conducted by methane oxidizing bacteria that act as a biofilter using methane as their energy and carbon source. Since their first discovery in 1906, methanotrophs have been studied using a complementary array of methods. One of the most used molecular methods involves PCR amplification of the functional gene marker for the diagnostic of copper and iron containing particulate methane monooxygenase. To investigate the diversity of methanotrophs and to extend their possible molecular detection, we designed a new set of degenerate methane monooxygenase primers to target an 850 nucleotide long sequence stretch from *pmoC* to *pmoA.* The primers were based on all available full genomic *pmoCAB* operons. The newly designed primers were tested on various pure cultures, enrichment cultures and environmental samples using PCR. The results demonstrated that this primer set has the ability to correctly amplify the about 850 nucleotide long *pmoCA* product from *Alphaproteobacteria*, *Gammaproteobacteria*, *Verrucomicrobia* and the NC10 phyla methanotrophs. The new primer set will thus be a valuable tool to screen ecosystems and can be applied in conjunction with previously used *pmoA* primers to extend the diversity of currently known methane-oxidizing bacteria.

**Electronic supplementary material:**

The online version of this article (doi:10.1186/s13568-017-0466-2) contains supplementary material, which is available to authorized users.

## Introduction

Methane is the second most important greenhouse gas contributing to about 20% of global warming (Intergovernmental Panel on Climate Change 2014). The global methane budget is estimated to be around 600 Tg a^−1^ (Dubey 2001) which is dominated by biogenic sources, where natural wetlands (23%), and rice fields (21%) (Frenzel 2000) account for almost half of the total budget (Chen and Prinn 2005). Methanogenic archaea are assumed to be the sole producers of methane that reside in these environments (Cicerone and Oremland 1988; Conrad et al. 1999; Joulian et al. 1997). These archaea are also present in waste treatment systems, intestines of ruminants and termites and landfills acting as additional CH_4_ sources. Therefore, microbial methanogenic activity is responsible for nearly 75% of the methane emitted to the atmosphere (Chen and Prinn 2005).

This process, is however, vastly mitigated by methanotrophic microorganisms that oxidize a large part of the produced CH_4_ (Cappelletti et al. 2016; Crevecoeur et al. 2015; Dumont and Murrell 2005; Reeburgh et al. 1993; Oshkin et al. 2014). It has been estimated that of the primary productivity, roughly 1% ends up in CH_4_; half of which is emitted into the atmosphere while the other half is consumed by methanotrophs (Reeburgh and Whjalen 2007; Aronson et al. 2013). While anaerobic methane-oxidizing archaea consume more than 75% of the CH_4_ produced in certain marine sediments (Reeburgh and Whjalen 2007; Beal et al. 2009; Egger et al. 2014), aerobic methane-oxidizing bacteria (MOB) that live at the interface between anoxic and oxic zones in marine environments (Bender and Conrad 1992; Lüke et al. 2016; Padilla et al. 2016), freshwater wetlands and rice fields (Lüke et al. 2014) have been estimated to consume up to 90% of the CH_4_ produced in these environments (Hanson and Hanson 1996). Alpha- and gammaproteobacterial methanotrophs have further been shown to be dominant methane consumers in acidic peatlands (Esson et al. 2016; Deng et al. 2013; Putkinen et al. 2014). Since their discovery over 100 years ago (Söhngen 1906), many aspects of methanotrophic bacteria have been studied (Whittenbury et al. 1970; Bédard and Knowles 1989; Hanson and Hanson 1996; Lidstrom 2006; Trotsenko and Murrell 2008). At the moment, several groups of aerobic bacteria are known that convert methane by means of a copper- and/or iron-containing enzyme called methane monooxygenase (MMO) (Murrell et al. 2000). Methanotrophic archaea play a prominent role in the anaerobic oxidation of methane and use methyl coenzyme-M reductase (MCR) (Knittel and Boetius 2009; Haroon et al. 2013; Welte et al. 2016).

Two different forms of MMO exist: a soluble MMO (sMMO) encoded by *mmoX*, *mmoY* and *mmoZ* and a particulate MMO encoded by *pmoCAB* (Lieberman and Rosenzweig 2005). The membrane bound particulate methane monooxygenase (pMMO) catalyzes the hydroxylation of methane. It exists in virtually all methanotrophs while sMMO has only been shown in certain genera such as *Methylococcus*, *Methylosinus*, *Methylocystis*, *Methylomonas* and *Methylocella* (Murrell et al. 2000). The more recent discovery of *Methylocella silvestris* (Crombie and Murrell, 2014), *Methyloferula stellata* (Dedysh et al. 2015), and *Methylocella palustris* (Dedysh et al. 2000) has illustrated that some MOB do indeed possess only sMMO and would not be targeted in pMMO-focused molecular studies (Dunfield et al. 2003; Dedysh et al. 2000; Vorobev et al. 2011; Vekeman et al. 2016a). pMMO belongs to the ammonia monooxygenase superfamily and has been shown to be of high biogeochemical and chemical relevance (Bédard and Knowles 1989; Hakemian and Rosenzweig 2007). This is due to the tight correlation that exists between this family and the globally important methane and nitrous oxide fluxes (Conrad 1996). This makes copper containing (Cu) MMO genes extremely useful markers in biological feedback studies looking at global climate change (Singh et al. 2010). Moreover, PCR-based environmental surveys have identified the ecological distribution and relevance of CuMMO-containing organisms correlated to gas flux, land use and climatic conditions (Coleman and the references within 2012). It has also been postulated that this group of enzymes could be correlated to processes other than methanotrophy and ammonia oxidation such as butane-oxidation (Coleman et al. 2012; Crombie and Murrell 2014). Therefore molecular approaches, such as PCR with specific primer sets are a fast and convenient method to screen for the diversity of such enzymes in various environments (Murrell et al. 1998; Mitsumori et al. 2002; Siljanen et al. 2012).

The crystal structure of pMMO has been determined to a resolution of 2.8 Å from *Methylococcus capsulatus* (Bath) and the enzyme has been found to be a trimer with an α_3_β_3_ɣ_3_ polypeptide arrangement (Lieberman and Rosenzweig 2005). The PmoA subunit contains non-heme iron in its center and for long was proposed to be the site of substrate hydroxylation. The soluble PmoB subunit hosts two metal centers, modelled as mononuclear copper and dinuclear copper, while a third metal center occupied by zinc is located within the membrane (Lieberman and Rosenzweig 2005).

Molecular surveys showed that MOB are present, amongst others, in natural wetlands (Costello et al. 2002; Samad and Bertilsson 2017), marine ecosystems (Vekeman et al. 2016b), permafrost thaw ponds (Crevecoeur et al. 2015), peatlands (Lau et al. 2015) and flooded rice-fields (Krüger et al. 2001; Lüke et al. 2009; Balasubramanian and Rosenzweig 2007; Zheng et al. 2008). Since pMMO was initially assumed to be present in all methane oxidizing bacteria, it has been used in molecular approaches to investigate methanotrophic diversity (Semrau et al. 1995; Holmes et al. 1999; Chi et al. 2012; Saidi-Mehrabad et al. 2013). More specifically *pmoA,* coding for the beta subunit of pMMO, was found to be highly conserved and as a result used as a functional gene marker (Holmes et al. 1995a, b; Bourne et al. 2001; Costello et al. 2002; Kolb et al. 2003; Luesken et al. 2011b; Wang et al. 2017).

In addition, *pmoA* amplicon pyrosequencing has been used to look at methanotrophic diversity in depth (Kip et al. 2011; Lüke and Frenzel 2011; Han and Gu 2013; Knief 2015). For all the PCR based methods, the used primers unfortunately do not encompass all different phyla of MOB to the same extent (Bergmann et al. 2011) nor do they cover new phyla such as *Verrucomicrobia* (Sharp et al. 2014; Erikstad and Birkeland 2015) and NC10. In the latter cases, more phylum specific primers had to be designed to investigate the presence of ‘*Candidatus Methylomirabilis oxyfera’* in various ecosystems (Luesken et al. 2011b). Recently several genomes of different MOB have been sequenced by the Omega consortium (Khmelenina et al. 2013; Kits et al. 2013; Khadem et al. 2012; Stephenson et al. 2017) and thus a much larger gene dataset is now available to design new primers to potentially cover a larger methanotroph diversity. Here we introduce a new set of degenerate primers that can be used to examine the diversity of MOB in various environments with the potential ability to target all presently known methanotrophic phyla. The new primers have the capability to target *pmoC* and *pmoA* and the intergenic region in between those genes. Application of the primers to various ecosystem resulted in the detection of *pmoCA* of *Alphaproteobacteria*, *Gammaproteobacteria*, *Verrucomicrobia* and NC10 within their respective habitats. Neither ammonia oxidizers, nor the recently discovered comammox (van Kessel et al. 2015; Pjevac et al. 2016) were detected with these primers. Furthermore, since the binding sites of the primers immediately flank the intergenic region between the genes *pmoC* and *pmoA*, they generate MOB lineage specific fragments. This unique property could be used in high throughput sequence analysis experiments for recovering diverse lineages in further environmental studies.

## Materials and methods

### Construction of *pmoCAB* operon database and primer design

A total of 83 different full genomic methane monooxygenase along with the isoenzyme PXM and ammonia monooxygenase gene sequences available on MaGe were downloaded (Vallenet et al. 2006; Sievers and Higgins 2014). This included *Alpha*-, *Gamma*-, and *Betaproteobacteria* (AOB), *Verrucomicrobia*, NC10, *Mycobacterium*, *Nocardia*, SAR cluster, divergent PXM operon and second operons from *Methylocystis parvus* OBBP, *Methylocystis* sp. BN69, *Methylosinus* sp. LW3, and *Methylosinus* sp. LW4 (Table 1). The genes were aligned in *pmoCAB* operon configuration. In cases where an organism’s genome contained more than one copy of the operon, all copies were included in the pipeline. Sequences were aligned using MUSCLE (Edgar 2004) and the alignment was imported into ARB (Ludwig et al. 2004). Nucleotide sequences were translated into protein sequences and phylogenetic trees were constructed based on the amino acid sequences. Furthermore, using the ‘Probe’ tool, primers that were capable of covering all (or as much as possible) phyla were designed within ARB. The parameters for the primer design were: 18 nucleotides in length, GC content of 50–70%, and minimum group coverage of at least 50%. Furthermore, the primers were made specific to MOB so that they had more than five mismatches with ammonium monooxygenase *amo* gene sequences of ammonia oxidizing bacteria (AOB).

**Table 1:**
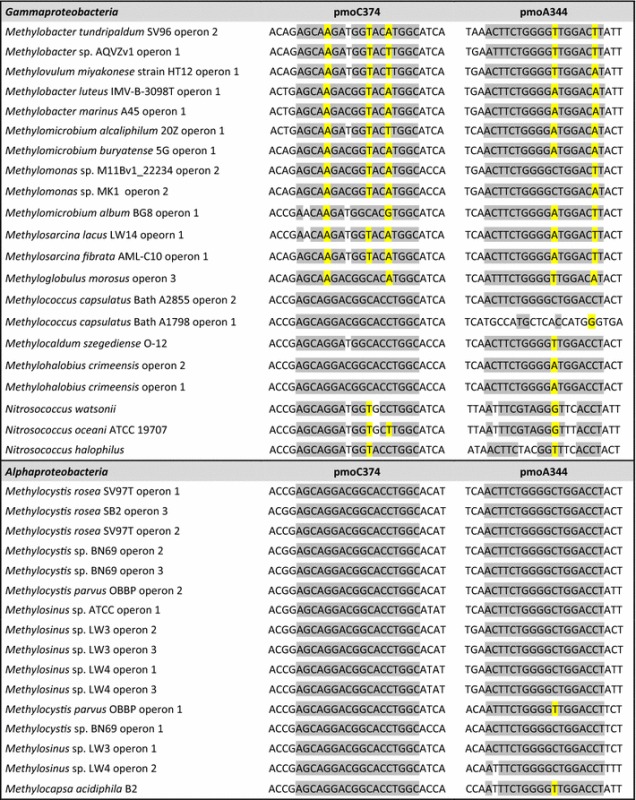

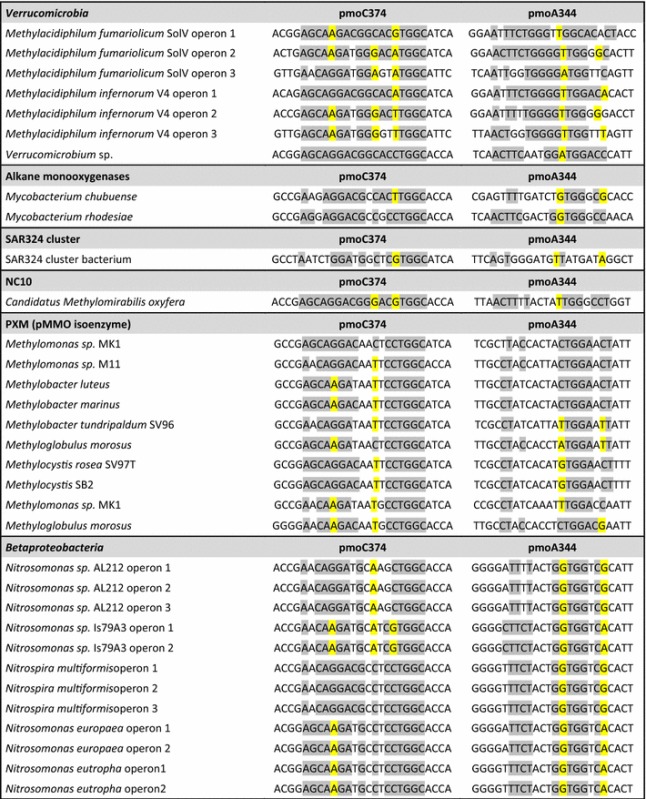
Aligment of the new pmoCA primers on all the available genomic sequences from different phyla. Wobble positions are shown in yellow

A set of primers covering *pmoC*, the intergenic region, and *pmoA* were ultimately designed (Table 2) and ordered from Biolegio (Nijmegen, the Netherlands). The forward primer, called pmoC374, with the reverse primer, called pmoA344 resulted in product length of roughly 850 base pairs (bp) (Table 3). There are slight variations between different lineages. This is caused by variation in on average, 120 bp long intergenic region between *pmoC* and *pmoA*.

**Table 2:** Comparison of targeting ability between two newly designed degenerate primers and pmoA189

Phylum	pmoC374				pmoA344				pmoA189			
	Mismatches				Mismatches				Mismatches			
	0	1	2	3	0	1	2	3	0	1	2	3
*Gammaproteobacteria*	10/18	18/18	18/18	18/18	16/18	18/18	18/18	18/18	7/18	18/18	18/18	18/18
*Alphaproteobacteria*	16/16	16/16	16/16	16/16	14/16	16/16	16/16	16/16	16/16	16/16	16/16	16/16
*Verrucomicrobia*	3/7	5/7	5/7	6/7	0/7	0/7	1/7	3/7	0/7	0/7	0/7	3/7
NC10	1/1	1/1	1/1	1/1	0/1	0/1	0/1	0/1	1/1	1/1	1/1	1/1

Percent sequence coverage of all *pmoCAB* available sequences within each phylum were calculated by looking at how many sequences each primer could target. Targeting ability is also shown for zero, one, two and three mismatches within each primer

**Table 3:** The new pMMO primers designed based on aligned pmoC, A, and B compared to pmoA189

Primers	Sequence	MT	%GC
PmoC374	5′-AGCARGACGGYACNTGGC-3′	42,9	56
PmoA189	5′-GGNGACTGGGACTTCTGG-3′	40,3	56
PmoA344	5′-ANGTCCAHCCCCAGAAGT-3′	42,9	50

*MT* melting temperature, *%GC* GC content in percentage

### DNA extraction and PCR conditions

Total DNA was extracted from methanotrophic pure and enrichment cultures and from various environmental samples. Table 4 provides an overview on the cultures and samples used in this study. DNA was extracted using the PowerSoil^®^ DNA Isolation Kit from MO BIO Laboratories (Carlsbad CA, USA) following the protocol of the manufacturer. The primers were tested using polymerase chain reaction (PCR), gradient PCR, touchdown PCR and nested PCR on all of the samples. The optimized protocol consisted of initial denaturation step at 96 °C for 5 min, followed by 35 cycles at 96 °C for 1 min, annealing at 55 °C for 1 min and elongation at 72 °C for 2 min. The final elongation step was done for 10 min at 72 °C.

**Table 4:** Over view of the strains, enrichment culture and environmental samples tested in this study to detect *pmoCA* gene sequences

Name/sample	Description	Origin/location reference
*Methylocystis rosea*	Pure culture *Alphaproteobacteria*	DSMZ 17621
*Methylosinus sporium*	Pure culture *Alphaproteobacteria*	DSMZ 17706
*Methylomonas lenta*	Pure culture *Gammaproteobacteria*	Hoefman et al. 2014
*Methyloacidimicrobium fagopyrum 3C*	Pure culture *Verrucomicrobia*	van Teeseling et al. 2014
*Methyloacidiphilum fumariolicum SolV*	Pure culture *Verrucomicrobia*	Pol et al. 2007
*Methylomirabilis oxyfera* (DAMO)	Enrichment culture NC10 phylum	Ooijpolder, NL Ettwig et al. 2008
Sludge from waste water treatment plant (WW)	Environmental sample	Lieshout, NL Luesken et al. (2011a, b)
Bulk soil form paddy field (BS)	Environmental sample	Vercelli, Italy Vaksmaa et al. (2016)
Rhizosphere of rice plants (ROOT)	Environmental sample	Vercelli, Italy Vaksmaa et al. (2016)
Enrichment culture with paddy field soil (RV)	Enrichment culture	Vaksmaa et al. (2016)
Volcanic mud (VM)	Environmental sample	Campi Flegrei caldera, Italy Pol et al. (2014)

Excision from gel after gel electrophoresis, purification, ligation and transformation of the amplified PCR products were done following the protocol described by Luesken et al. 2011a. At least 20 random clones were picked for each environmental sample in a blue-white screening and the plasmids were isolated for each PCR product with the GeneJet Miniprep Kit (Fermentas, Vilnus, Lithuania). The samples were sent to BaseClear (Leiden, the Netherlands) for sequencing of the cloned product using M13 forward primer (Luesken et al. 2011a).

### Sequence analysis

The resulting sequences were checked for quality using Chromas Lite 2.1.1.0 (Technelysium Pty Ltd). Once erroneous sequences were removed, the results were blasted (BLASTx) using the publically available tools on National Center for Biotechnology Information (NCBI). Sequences matching with AMO superfamily were imported into ARB, translated into protein sequences and aligned to the previously mentioned *pmoCAB* operon dataset using ARB built-in aligner tools. Phylogenetic tree construction was performed on the amino acid alignment using maximum parsimony and maximum likelihood methods with bootstrapping of 100 times. Consensus sequences based on the fraction and frequency of residues at a specific alignment position within *pmoC* from all sequences were used to generate the tree.

Sequences are deposited in Genbank with Accession Numbers KY883458–KY883555 (Additional file 1: Table S1).

## Results

The design of new primers was obtained by using all available *pmoCAB* operon sequences from MaGe. Interestingly, *pmoB* contained no conserved sequence stretch as a potential primer target site. Looking at the full operons, the only conserved regions resided within *pmoC* and *pmoA*. A new region at the nucleotide position 374 within the PmoC subunit of *Methylococcus capsulatus* (Bath), as a reference, was found to be highly conserved amongst all the phyla tested in this experiment. The forward primer binding site encodes for a glutamine residue at 126th base within the crystal structure of *pmoC* anchored to the membrane in *Methylococcus capsulatus* (Bath) whereas the reverse primer binding site encodes a phenylalanine residue at 107th base within *pmoA*. Our newly designed forward primer was compared to Holmes’ forward primer and the results are shown in Tables 1 and 2. As the tables illustrate, with zero mismatches, pmoC374 is able to target three out of the seven available sequences from *Verrucomicrobia*. If a single mismatch is allowed, five out of the seven sequences from *Verrucomicrobia* are targeted by pmoC374 whereas pmoA189 (Holmes et al. 1995a, b) with one mismatch still does not target any verrucomicrobial pMMO gene. The details of the novel primer set with regards to number of mismatches are listed in Table 2.

**Fig. 1:**
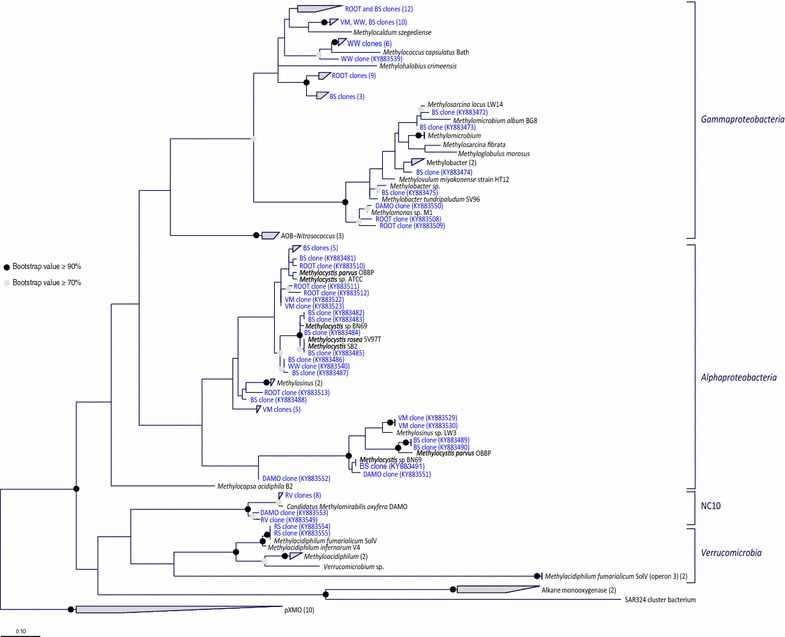
Representing available pMMO sequences including the sequence obtained in this study. The tree was constructed using consensus sequence, based on the fraction and frequency of residues at an alignment position chosen within *pmoC* using both ARB’s PHYML (amino Acids) tool within the maximum likelihood method and Phylip PROTPARS within the maximum parsimony method. Since the two trees were highly similar, only maximum likelihood is shown here. Due to size limitation, the tree is partially collapsed for an easier illustration and pXMO is used as the outgroup instead of AOB sequences that are omitted from this figure. The tree was built with 100 bootstraps and the ranges of values are shown with the respective *colored circles* at each node. Clone sequences with their respective accession numbers are *highlighted in blue* and the *numbers in the brackets* correspond to the number of sequences within a group. *Gammaproteobacteria, Alphaproteobacteria,* NC10 and *Verrucomicrobia* are clearly distinguished in the figure. Origin of clones: *BS* bulk soil, *ROOT* rhizosphere, *VM* volcanic mud, *WW* waste water sludge, *RV* bioreactor enrichment from vercelli, *RS Methylacidiphilum fumariolicum* SolV, *DAMO Methylomirabilis oxyfera* enrichment culture

Initially, pmoA189 target region was thought to be a good matching reverse primer, however, a secondary conserved region at the 334th position within the *pmoA* gene was found. The pmoC374 with pmoA344 combination yielded a PCR product of the correct size in the samples tested, while the same could not always be observed when it was used in combination with pmoA189. In Table 1 and 2, it can be observed that pmoA344 has the ability to target 17 out of the 19 sequences belonging to *Gammaproteobacteria* with zero mismatches. Based on sequence information, pmoA334 does not have the ability to target NC10 phylum and it needs two or more mismatches to target species belonging to *Verrucomicrobia*. However, this primer improved the ability to target both *Verrucomicrobia* and the NC10 phyla in our study when pure isolates were used as positive control in the PCR reaction. The resulting sequences from the various enrichment cultures and environmental samples are depicted in Fig. 1.

The *pmoCA* sequences obtained from the paddy field sample were closely related to well-known genera including *Methylosinus*, *Methylocystis*, *Methylococcus*, *Methylocaldum*, *Methylohalobius*, *Methylomicrobium*, *Methylobacter* and *Methylomonas*. Furthermore, the *pmoCA* of pure cultures of *Methylocystis rosea* and *Methylosinus sporium* belonging to *Alphaproteobacteria* and *Methylomonas lenta* (Hoefman et al. 2014) belonging to *Gammaproteobacteria* could all be amplified with the new primer set. From previous studies, two isozymes of pMMO with various methane oxidation kinetics were found to be present in *Methylocystis* sp. strain SC2 (Baani and Liesack 2008), the new primers also amplified the second *pmoCA* in DNA extracted from the paddy soil. *Methylocaldum*- and *Methylococcus*-like species were also found in Waste Water samples. Furthermore, both alpha- and gammaproteobacterial *pmoCA* were found in the volcanic mud sample. Lastly, the *pmoCA* of the verrucomicrobial methanotroph *Methylacidiphilum fumarolicum* SolV could be amplified as well from a pure culture (Fig. 1).

In our experiment, only the *Verrucomicrobia* pMMO sequence most closely related to the ones in *Alphaproteobacteria* and *Gammaproteobacteria* could be detected. The new primer set was also used on a pure mesophilic *Verrucomicrobia* strain *Methyloacidimicrobium fagopyrum 3C* resulting in gene product of the correct size and gene sequence. The primers do not amplify sequences related to the *pmoC3* group. In both anoxic enrichment cultures (DAMO and RV) tested, the *pmoCA* of NC10 phylum bacterium *Methylomirabilis oxyfera* could be amplified (Fig. 1). In the case of *Methylomonas lenta* that does contain the genes for pXMO, only *pmoCA* gene sequences were detected, while the pXMO was not amplified. Lastly, no AMO (ammonia monooxygenase), PXM (alternative methane monooxygenase) or the recently discovered comammox amo were targeted nor amplified with this primer set in any of the environmental samples or the negative controls used in this study.

## Discussion

In the era of ‘omics’, molecular approaches using either specific or degenerate primers are still of high importance, especially in ecological studies where many samples need to be investigated or screened. They allow for a quick and robust detection of uncultivated microbes and aid in hypothesizing the community structure and the key processes that occur in certain environments, at the molecular level. As our knowledge and understanding of these environments expands, the tools that are used to investigate also need to be updated. More specifically, identification of the diverse organisms responsible for the oxidation of methane within various environments will help to better understand the key players involved in the methane cycle and evaluate their potential effectiveness as a biological methane filter. The currently available *pmoA* based primers are over 10 years old and since known MOB diversity has since been extended, a novel primer set with broader amplification ability would be highly beneficial in molecular studies. It is also important to distinguish between copper monooxygenases belonging to the AMO superfamily to ensure the detection of MOB and not AOB or the more recently discovered comammox (van Kessel et al. 2015; Pjevac et al. 2016; Pinto et al. 2015).

The use of all available *pmoCAB* operon sequences from MaGe allowed for the design of new primers (Table 1). Interestingly *pmoB*, which in previous studies has been suggested as the active site of the methane monooxygenase enzyme (Culpepper and Rosenzweig 2012; Lieberman and Rosenzweig 2005) contained no conserved sequence stretch as a potential primer target site. The only conserved regions that could be observed resided within *pmoC* and *pmoA*, both of which encode for primarily membrane bound subunits (Lieberman and Rosenzweig 2005). Overall, PmoA is by far the most conserved subunit of this enzyme. Since for long it was thought to be the catalytic subunit as well, primers were designed based on this gene and have since become the academic standard in this line of research and used to date in many studies (Lüke and Frenzel 2011; Rastogi et al. 2009; Kip et al. 2011). However, due to the two mismatches that occur at the 10th position within *pmoA* target region, previously unknown phyla (i.e. *Verrucomicrobia* or NC10) remain undetected and demand the design of phylum specific primers (Luesken et al. 2011b). This variation in sequence identity is also one of the reasons why this study focused on the whole *pmoCAB* operon instead of the PmoA subunit alone (Table 2).

Previous studies have looked into analysis of MOB community in rice fields by targeting 16S rRNA, pMMO and methanol dehydrogenase (Henckel et al. 1999) and observed a large variety of MOB. The new primer set used in this study was also able to detect a wide array of *pmoCA* sequences from both the bulk soil as well as the rhizosphere of an Italian rice paddy field, a waste water treatment sample, and volcanic mud samples. Further in anoxic *Methylomirabilis oxyfera* enrichment cultures started with paddy field or Ooijpolder sediment, many different *pmoCA* sequences could be retrieved (Fig. 1).

Furthermore, the *pmoCA* of the verrucomicrobial methanotroph *Methylacidiphilum fumarolicum* SolV could be amplified. This strain contains three complete *pmoCAB* operon structures that resemble the one observed in proteobacterial methanotrophs, plus a fourth *pmoC* copy. As expected, the primers do not amplify sequences related to the *pmoC3* group as it is further downstream in the genome and the primers do not bind there.

Most sequences from the Waste Water Treatment Plant biomass used in this study were closely related to *Methylococcus* genus as was previously observed (Luesken et al. 2011a). Lastly, no AMO (ammonia monooxygenase), PXM (alternative methane monooxygenase) or the recently discovered commamox amo were targeted nor amplified with this primer set in any of the environmental samples which is an indication of the specificity. However, with some modification of the primer sequence, the same or similar sites can be used to only target AOB instead of MOB (Pjevac et al. 2016; Wang et al. 2017).

This study illustrates that when primer pmoC374 was used in combination with pmoA344, PCR amplification yielded the correct gene product from various environmental samples and MOB strains. Such observation could not be made when pmoA189 was used as the reverse primer. At times, there were multiple bands that occurred at the expected size within the gel. When each band was excised from the gel, all corresponded to the correct product. Since the *pmoCA* sequence covers the intergenic region, the slightly different nucleotide length observed in the PCR product is possibly due to the variation that exists in this region. This was more apparent when environmental samples were used as opposed to pure isolates, which further supports our hypothesis.

The obtained results expand our knowledge with regard to primer target ability based solely on in silico coverage as supposed to experimental results, since the new targeting sites would not be desirable due to occurring mismatches. Furthermore, the new pMMO primer set was able to amplify the correct product and sequence from all currently known methanotrophic phyla. If used in conjunction with Holmes’ forward primer, the resulting product could be used in future next generation sequencing studies for a more extensive look at the bacterial community structure. The concurrent use of this primer set along with ones based solely on *pmoA* would allow for a much lesser bias when it comes to studies that look at the general diversity of the methanotrophic community within various environments. It also permits for the simultaneous detection of *Alphaproteobacteria*, *Gammaproteobacteria*, *Verrucomicrobia* and NC10 phyla with broader sequence variation.

## Additional file

**Additional file 1: Table S1.** Sequences belonging to each environmental samples and their respective accession numbers from Genbank.

## Abbreviations

MOB: methane oxidizing bacteria
MMO: methane monooxygenase
MCR: methyl coenzyme-M reductase
sMMO: soluble methane monooxygenase
pMMO: particulate methane monooxygenase
CuMMO: copper containing methane monooxygenase
PCR: polymerase chain reaction
rRNA: ribosomal ribonucleic acid
