# Supplementary material for: Survey of methanotrophic diversity in various ecosystems by degenerate methane monooxygenase gene primers
Source: AMB Express. 2017 Aug 23;7:162. doi: 10.1186/s13568-017-0466-2 (PMC5567572; doi:10.1186/s13568-017-0466-2)
Supplement: Supplementary file 1 — Additional file 1: Table S1. Sequences belonging to each environmental samples and their respective accession numbers from Genbank. [file 13568_2017_466_MOESM1_ESM.docx]

AMB Express

Survey of methanotrophic diversity in various ecosystems by degenerate methane monooxygenase gene primers

Mohammad Ghashghavi, Mike S.M. Jetten, Claudia Lüke

Department of Microbiology, IWWR institude, Radboud University, Heijendaalsweg 135

6525 AJ Nijmegen, the Netherlands

Email: mghash@science.ru.nl

Tel: 024 365 2657

Table S1: Sequences belonging to each environmental samples and their respective accession numbers from Genbank. BS = Bulk Soil, ROOT = Rhizosphere, VM = Volcanic Mud, WW = Waste Water sludge, RV = Bioreactor enrichment from Vercelli, DAMO = Bioreactor enrichment from Ooijplder, SolV = *Methylacidiphilum fumariolicum* SolV bioreactor.

| **Sequence number** | **Clone ID** | **Accession number** |
| --- | --- | --- |
| Seq1 | BS1 | KY883458 |
| Seq2 | BS2 | KY883459 |
| Seq3 | BS3 | KY883460 |
| Seq4 | BS4 | KY883461 |
| Seq5 | BS5 | KY883462 |
| Seq6 | BS6 | KY883463 |
| Seq7 | BS7 | KY883464 |
| Seq8 | BS8 | KY883465 |
| Seq9 | BS9 | KY883466 |
| Seq10 | BS10 | KY883467 |
| Seq11 | BS11 | KY883468 |
| Seq12 | BS12 | KY883469 |
| Seq13 | BS13 | KY883470 |
| Seq14 | BS14 | KY883471 |
| Seq15 | BS15 | KY883472 |
| Seq16 | BS16 | KY883473 |
| Seq17 | BS17 | KY883474 |
| Seq18 | BS18 | KY883475 |
| Seq19 | BS19 | KY883476 |
| Seq20 | BS20 | KY883477 |
| Seq21 | BS21 | KY883478 |
| Seq22 | BS22 | KY883479 |
| Seq23 | BS23 | KY883480 |
| Seq24 | BS24 | KY883481 |
| Seq25 | BS25 | KY883482 |
| Seq26 | BS26 | KY883483 |
| Seq27 | BS27 | KY883484 |
| Seq28 | BS28 | KY883485 |
| Seq29 | BS29 | KY883486 |
| Seq30 | BS30 | KY883487 |
| Seq31 | BS31 | KY883488 |
| Seq32 | BS32 | KY883489 |
| Seq33 | BS33 | KY883490 |
| Seq34 | BS34 | KY883491 |
| Seq35 | ROOT1 | KY883492 |
| Seq36 | ROOT2 | KY883493 |
| Seq37 | ROOT3 | KY883494 |
| Seq38 | ROOT4 | KY883495 |
| Seq39 | ROOT5 | KY883496 |
| Seq40 | ROOT6 | KY883497 |
| Seq41 | ROOT7 | KY883498 |
| Seq42 | ROOT8 | KY883499 |
| Seq43 | ROOT9 | KY883500 |
| Seq44 | ROOT10 | KY883501 |
| Seq45 | ROOT11 | KY883502 |
| Seq46 | ROOT12 | KY883503 |
| Seq47 | ROOT13 | KY883504 |
| Seq48 | ROOT14 | KY883505 |
| Seq49 | ROOT15 | KY883506 |
| Seq50 | ROOT16 | KY883507 |
| Seq51 | ROOT17 | KY883508 |
| Seq52 | ROOT18 | KY883509 |
| Seq53 | ROOT19 | KY883510 |
| Seq54 | ROOT20 | KY883511 |
| Seq55 | ROOT21 | KY883512 |
| Seq56 | ROOT22 | KY883513 |
| Seq57 | VM1 | KY883514 |
| Seq58 | VM2 | KY883515 |
| Seq59 | VM3 | KY883516 |
| Seq60 | VM4 | KY883517 |
| Seq61 | VM5 | KY883518 |
| Seq62 | VM6 | KY883519 |
| Seq63 | VM7 | KY883520 |
| Seq64 | VM8 | KY883521 |
| Seq65 | VM9 | KY883522 |
| Seq66 | VM10 | KY883523 |
| Seq67 | VM11 | KY883524 |
| Seq68 | VM12 | KY883525 |
| Seq69 | VM13 | KY883526 |
| Seq70 | VM14 | KY883527 |
| Seq71 | VM15 | KY883528 |
| Seq72 | VM16 | KY883529 |
| Seq73 | VM17 | KY883530 |
| Seq74 | WW1 | KY883531 |
| Seq75 | WW2 | KY883532 |
| Seq76 | WW3 | KY883533 |
| Seq77 | WW4 | KY883534 |
| Seq78 | WW5 | KY883535 |
| Seq79 | WW6 | KY883536 |
| Seq80 | WW7 | KY883537 |
| Seq81 | WW8 | KY883538 |
| Seq82 | WW9 | KY883539 |
| Seq83 | WW10 | KY883540 |
| Seq84 | RV1 | KY883541 |
| Seq85 | RV2 | KY883542 |
| Seq86 | RV3 | KY883543 |
| Seq87 | RV4 | KY883544 |
| Seq88 | RV5 | KY883545 |
| Seq89 | RV6 | KY883546 |
| Seq90 | RV7 | KY883547 |
| Seq91 | RV8 | KY883548 |
| Seq92 | RV9 | KY883549 |
| Seq93 | DAMO1 | KY883550 |
| Seq94 | DAMO2 | KY883551 |
| Seq95 | DAMO3 | KY883552 |
| Seq96 | DAMO4 | KY883553 |
| Seq97 | SolV | KY883554 |
| Seq98 | SolV | KY883555 |
